# 5-(Adamantan-1-yl)-*N*-methyl-1,3,4-thia­diazol-2-amine

**DOI:** 10.1107/S1600536813009033

**Published:** 2013-04-10

**Authors:** Abdul-Malek S. Al-Tamimi, Ahmed M. Alafeefy, Ali A. El-Emam, Seik Weng Ng, Edward R. T. Tiekink

**Affiliations:** a Department of Pharmaceutical Chemistry, College of Pharmacy, Salman bin Abdulaziz University, Alkharj 11942, Saudi Arabia; b Department of Pharmaceutical Chemistry, College of Pharmacy, King Saud University, Riyadh 11451, Saudi Arabia; c Department of Chemistry, University of Malaya, 50603 Kuala Lumpur, Malaysia; d Chemistry Department, Faculty of Science, King Abdulaziz University, PO Box 80203 Jeddah, Saudi Arabia

## Abstract

In the title compound, C_13_H_19_N_3_S, the methyl­amine substituent is coplanar with the thia­diazole ring to which it is attached [C—N—C—S torsion angle = 175.9 (2)°] and the amine H atom is *syn* to the thia­diazole S atom. Supra­molecular chains along [101], sustained by N—H⋯N hydrogen bonding, feature in the crystal packing.

## Related literature

For the biological activity of 1,3,4-thia­diazol-2-amine derivatives, see: Carvalho *et al.* (2008 ▶); Foroumadi *et al.* (1999 ▶), and of adamantane derivatives, see: Togo *et al.* (1968 ▶); El-Emam *et al.* (2004 ▶). For related structures, see: El-Emam *et al.* (2012 ▶); Almutairi *et al.* (2012 ▶). For the synthesis of the title compound, see: El-Emam & Lehmann (1994 ▶).
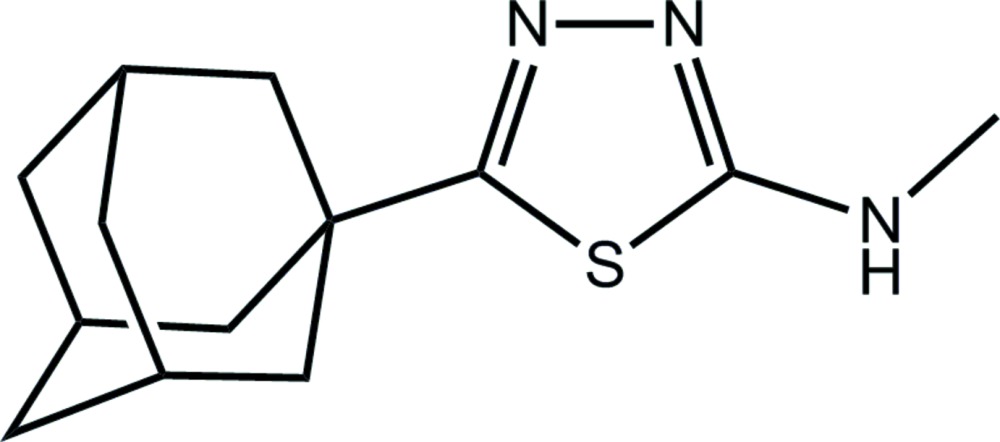


## Experimental

### 

#### Crystal data

C_13_H_19_N_3_S
*M* *_r_* = 249.37
Monoclinic, 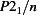

*a* = 10.4394 (12) Å
*b* = 13.0910 (13) Å
*c* = 10.8871 (15) Å
β = 118.008 (16)°
*V* = 1313.6 (3) Å^3^
*Z* = 4
Mo *K*α radiation
μ = 0.23 mm^−1^
*T* = 295 K
0.30 × 0.20 × 0.10 mm

#### Data collection

Agilent SuperNova Dual diffractometer with an Atlas detector
Absorption correction: multi-scan (*CrysAlis PRO*; Agilent, 2012 ▶) *T*_min_ = 0.887, *T*_max_ = 1.000
6791 measured reflections
3027 independent reflections
1975 reflections with *I* > 2σ(*I*)
*R*_int_ = 0.039

#### Refinement

*R*[*F* ^2^ > 2σ(*F* ^2^)] = 0.054
*wR*(*F* ^2^) = 0.144
*S* = 1.04
3027 reflections
159 parameters
1 restraint
H atoms treated by a mixture of independent and constrained refinement
Δρ_max_ = 0.24 e Å^−3^
Δρ_min_ = −0.22 e Å^−3^

### 

Data collection: *CrysAlis PRO* (Agilent, 2012 ▶); cell refinement: *CrysAlis PRO*; data reduction: *CrysAlis PRO*; program(s) used to solve structure: *SHELXS97* (Sheldrick, 2008 ▶); program(s) used to refine structure: *SHELXL97* (Sheldrick, 2008 ▶); molecular graphics: *ORTEP-3 for Windows* (Farrugia, 2012 ▶) and *DIAMOND* (Brandenburg, 2006 ▶); software used to prepare material for publication: *publCIF* (Westrip, 2010 ▶).

## Supplementary Material

Crystal structure: contains datablock(s) global, I. DOI: 10.1107/S1600536813009033/hg5305sup1.cif

Structure factors: contains datablock(s) I. DOI: 10.1107/S1600536813009033/hg5305Isup2.hkl

Supplementary material file. DOI: 10.1107/S1600536813009033/hg5305Isup3.cml

Additional supplementary materials:  crystallographic information; 3D view; checkCIF report

## Figures and Tables

**Table 1:** Hydrogen-bond geometry (Å, °)

*D*—H⋯*A*	*D*—H	H⋯*A*	*D*⋯*A*	*D*—H⋯*A*
N3—H3⋯N1^i^	0.87 (1)	2.15 (1)	3.021 (3)	179 (2)

Symmetry code: (i) 
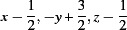
.

**Fig. 1.**
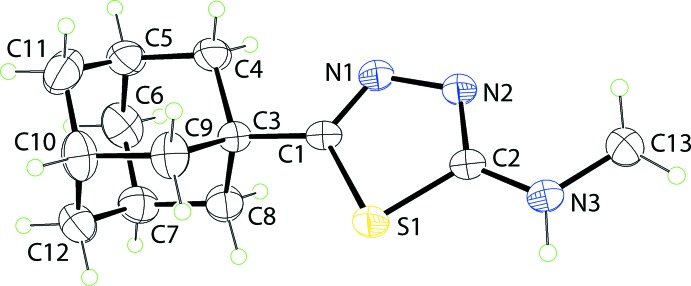
The molecular structure of (I) showing the atom-labelling scheme and displacement ellipsoids at the 35% probability level.

**Fig. 2.**
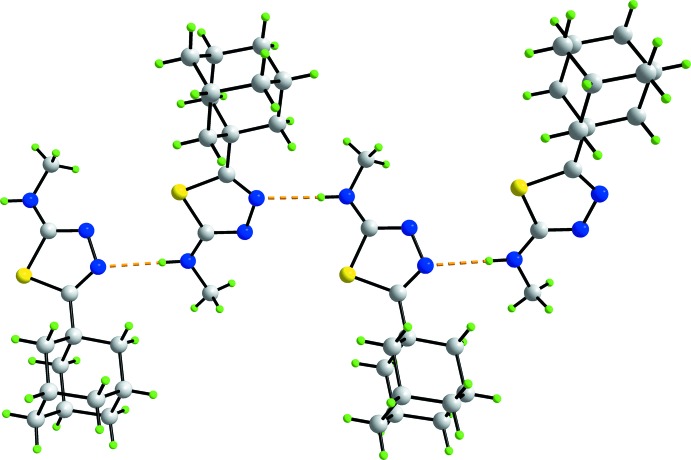
A view of the supramolecular chain in (I) sustained by N—H···N hydrogen bonds shown as orange dashed lines.

**Fig. 3.**
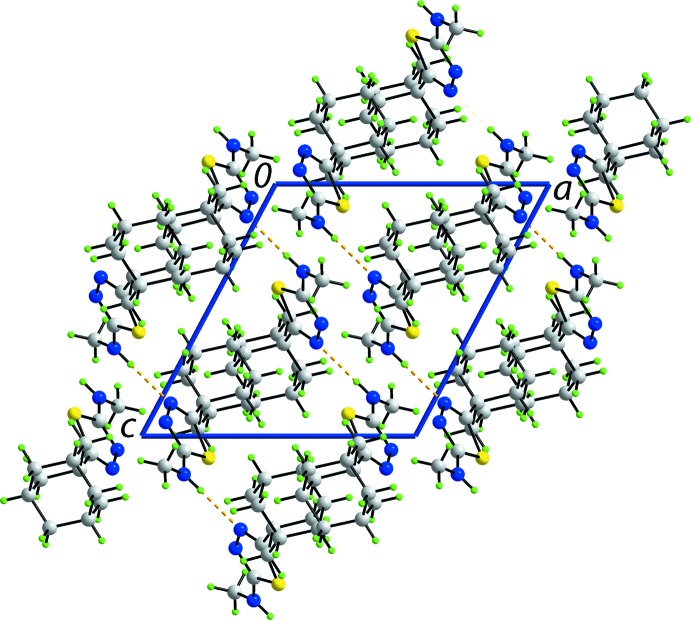
A view in projection down the b axis of the unit-cell contents for (I).

## supplementary crystallographic information

### Comment

Derivatives of adamantane have long been known for their diverse biological activities including anti-viral activity against influenza (Togo *et al.*, 1968) and HIV viruses (El-Emam *et al.*, 2004). Moreover, 1,3,4-thiadiazole derivatives were reported to exhibit marked anti-trypanosomal (Carvalho *et al.*, 2008) and anti-microbial activities (Foroumadi *et al.*, 1999). In continuation of our interest in the chemical and pharmacological properties of adamantane derivatives, and as part of on-going structural studies of these (El-Emam *et al.*, 2012; Almutairi *et al.*, 2012), we report herein the X-ray crystallographic data of the title compound, (I).

In (I), Fig. 1, the five-membered ring is planar (r.m.s. deviation = 0.009 Å) and the methylamine substituent is co-planar: the C13—N3—C2—S1 torsion angle is 175.9 (2)°. The amine-H atom is *syn* to the thiadiazole-S1 atom. N—H···N hydrogen bonds feature in the crystal packing, leading to supramolecular chains along [1 0 1], Fig. 2 and Table 1. Chains pack with no specific intermolecular interactions between them. Globally, the crystal structure comprises alternating layers of hydrophilic and hydrophobic regions, Fig. 3.

### Experimental

The title compound was prepared by dehydrative cyclization of 1-(1-adamantylcarbonyl)-4-methylthiosemicarbazide using sulfuric acid at room temperature for 24 h as previously described (El-Emam & Lehmann, 1994). Single crystals were obtained by slow evaporation from its CHCl_3_:EtOH solution at room temperature; *M*.pt: 441–443 K.

### Refinement

The C-bound H-atoms were placed in calculated positions [C—H = 0.96 to 0.98 Å, *U*_iso_(H) = 1.2 or 1.5*U*_eq_(C)] and were included in the refinement in the riding model approximation. The N-bound H-atom was refined with N—H = 0.88±0.01 Å.

### Crystal data

**Table:**

C_13_H_19_N_3_S	*F*(000) = 536
*M**_r_* = 249.37	*D*_x_ = 1.261 Mg m^−^^3^
Monoclinic, *P*2_1_/*n*	Mo *K*α radiation, λ = 0.71073 Å
Hall symbol: -P 2yn	Cell parameters from 1515 reflections
*a* = 10.4394 (12) Å	θ = 3.1–27.5°
*b* = 13.0910 (13) Å	µ = 0.23 mm^−^^1^
*c* = 10.8871 (15) Å	*T* = 295 K
β = 118.008 (16)°	Prism, colourless
*V* = 1313.6 (3) Å^3^	0.30 × 0.20 × 0.10 mm
*Z* = 4	

### Data collection

**Table:**

Agilent SuperNova Dual diffractometer with an Atlas detector	3027 independent reflections
Radiation source: SuperNova (Mo) X-ray Source	1975 reflections with *I* > 2σ(*I*)
Mirror monochromator	*R*_int_ = 0.039
Detector resolution: 10.4041 pixels mm^-1^	θ_max_ = 27.6°, θ_min_ = 3.1°
ω scan	*h* = −13→13
Absorption correction: multi-scan (*CrysAlis PRO*; Agilent, 2012)	*k* = −16→17
*T*_min_ = 0.887, *T*_max_ = 1.000	*l* = −13→14
6791 measured reflections	

### Refinement

**Table:**

Refinement on *F*^2^	Primary atom site location: structure-invariant direct methods
Least-squares matrix: full	Secondary atom site location: difference Fourier map
*R*[*F*^2^ > 2σ(*F*^2^)] = 0.054	Hydrogen site location: inferred from neighbouring sites
*wR*(*F*^2^) = 0.144	H atoms treated by a mixture of independent and constrained refinement
*S* = 1.04	*w* = 1/[σ^2^(*F*_o_^2^) + (0.0539*P*)^2^ + 0.1967*P*] where *P* = (*F*_o_^2^ + 2*F*_c_^2^)/3
3027 reflections	(Δ/σ)_max_ < 0.001
159 parameters	Δρ_max_ = 0.24 e Å^−^^3^
1 restraint	Δρ_min_ = −0.22 e Å^−^^3^

### Special details

**Table:**

Geometry. All e.s.d.'s (except the e.s.d. in the dihedral angle between two l.s. planes) are estimated using the full covariance matrix. The cell e.s.d.'s are taken into account individually in the estimation of e.s.d.'s in distances, angles and torsion angles; correlations between e.s.d.'s in cell parameters are only used when they are defined by crystal symmetry. An approximate (isotropic) treatment of cell e.s.d.'s is used for estimating e.s.d.'s involving l.s. planes.
Refinement. Refinement of *F*^2^ against ALL reflections. The weighted *R*-factor *wR* and goodness of fit *S* are based on *F*^2^, conventional *R*-factors *R* are based on *F*, with *F* set to zero for negative *F*^2^. The threshold expression of *F*^2^ > σ(*F*^2^) is used only for calculating *R*-factors(gt) *etc*. and is not relevant to the choice of reflections for refinement. *R*-factors based on *F*^2^ are statistically about twice as large as those based on *F*, and *R*- factors based on ALL data will be even larger.

### Fractional atomic coordinates and isotropic or equivalent isotropic displacement parameters (Å^2^)

**Table:**

	*x*	*y*	*z*	*U*_iso_*/*U*_eq_	
S1	0.21237 (6)	0.64435 (5)	0.41269 (6)	0.0480 (2)	
N1	0.45652 (19)	0.66351 (15)	0.62872 (19)	0.0446 (5)	
N2	0.43886 (18)	0.75355 (15)	0.55520 (19)	0.0455 (5)	
N3	0.2711 (2)	0.83210 (16)	0.3481 (2)	0.0514 (5)	
H3	0.1801 (12)	0.8326 (19)	0.2850 (19)	0.047 (7)*	
C1	0.3500 (2)	0.60016 (19)	0.5713 (2)	0.0398 (5)	
C2	0.3152 (2)	0.75433 (18)	0.4397 (2)	0.0404 (5)	
C3	0.3392 (2)	0.49988 (18)	0.6325 (2)	0.0398 (5)	
C4	0.4805 (3)	0.4770 (2)	0.7646 (3)	0.0614 (7)	
H4A	0.5016	0.5321	0.8308	0.074*	
H4B	0.5598	0.4725	0.7420	0.074*	
C5	0.4676 (3)	0.3761 (2)	0.8298 (3)	0.0698 (9)	
H5	0.5580	0.3627	0.9150	0.084*	
C6	0.3426 (3)	0.3824 (3)	0.8635 (3)	0.0744 (9)	
H6A	0.3359	0.3190	0.9062	0.089*	
H6B	0.3600	0.4373	0.9292	0.089*	
C7	0.2019 (3)	0.4013 (2)	0.7331 (3)	0.0614 (7)	
H7	0.1221	0.4047	0.7564	0.074*	
C8	0.2149 (3)	0.5035 (2)	0.6717 (3)	0.0566 (7)	
H8A	0.2337	0.5575	0.7391	0.068*	
H8B	0.1242	0.5188	0.5896	0.068*	
C9	0.3080 (3)	0.4115 (2)	0.5300 (3)	0.0605 (7)	
H9A	0.3848	0.4073	0.5040	0.073*	
H9B	0.2175	0.4242	0.4465	0.073*	
C10	0.2977 (3)	0.3098 (2)	0.5956 (3)	0.0682 (8)	
H10	0.2792	0.2540	0.5294	0.082*	
C11	0.1735 (3)	0.3176 (2)	0.6301 (3)	0.0675 (8)	
H11A	0.0840	0.3314	0.5459	0.081*	
H11B	0.1625	0.2533	0.6683	0.081*	
C12	0.4399 (3)	0.2912 (3)	0.7263 (4)	0.0807 (10)	
H12A	0.4358	0.2262	0.7671	0.097*	
H12B	0.5187	0.2882	0.7028	0.097*	
C13	0.3567 (3)	0.9237 (2)	0.3810 (3)	0.0619 (7)	
H13A	0.3160	0.9693	0.3028	0.093*	
H13B	0.4544	0.9068	0.4020	0.093*	
H13C	0.3570	0.9562	0.4602	0.093*	

### Atomic displacement parameters (Å^2^)

**Table:**

	*U*^11^	*U*^22^	*U*^33^	*U*^12^	*U*^13^	*U*^23^
S1	0.0374 (3)	0.0496 (4)	0.0422 (4)	−0.0049 (3)	0.0063 (3)	0.0015 (3)
N1	0.0365 (9)	0.0478 (12)	0.0403 (11)	0.0009 (9)	0.0105 (8)	0.0020 (9)
N2	0.0361 (9)	0.0460 (12)	0.0435 (11)	−0.0023 (9)	0.0096 (8)	0.0033 (10)
N3	0.0380 (10)	0.0503 (13)	0.0490 (13)	−0.0012 (10)	0.0063 (9)	0.0077 (11)
C1	0.0314 (10)	0.0487 (14)	0.0366 (12)	0.0015 (10)	0.0138 (9)	−0.0020 (11)
C2	0.0327 (10)	0.0455 (14)	0.0396 (12)	0.0020 (10)	0.0142 (9)	0.0001 (11)
C3	0.0366 (11)	0.0435 (13)	0.0388 (12)	0.0010 (10)	0.0171 (9)	0.0007 (11)
C4	0.0435 (13)	0.0650 (18)	0.0611 (17)	−0.0039 (13)	0.0125 (12)	0.0184 (14)
C5	0.0500 (14)	0.070 (2)	0.0689 (19)	−0.0026 (15)	0.0110 (14)	0.0242 (17)
C6	0.089 (2)	0.081 (2)	0.0559 (18)	−0.0179 (18)	0.0364 (17)	0.0018 (16)
C7	0.0560 (15)	0.073 (2)	0.0647 (18)	−0.0040 (14)	0.0364 (14)	0.0026 (15)
C8	0.0556 (14)	0.0574 (17)	0.0643 (17)	0.0008 (13)	0.0344 (13)	−0.0009 (14)
C9	0.0785 (18)	0.0535 (16)	0.0597 (17)	−0.0018 (14)	0.0408 (15)	−0.0035 (14)
C10	0.095 (2)	0.0474 (16)	0.070 (2)	−0.0086 (16)	0.0448 (18)	−0.0112 (15)
C11	0.0611 (16)	0.0589 (18)	0.075 (2)	−0.0128 (15)	0.0254 (15)	0.0020 (16)
C12	0.081 (2)	0.066 (2)	0.115 (3)	0.0235 (18)	0.062 (2)	0.034 (2)
C13	0.0534 (14)	0.0537 (17)	0.0642 (18)	−0.0072 (13)	0.0157 (13)	0.0063 (14)

### Geometric parameters (Å, º)

**Table:**

S1—C2	1.737 (2)	C6—H6B	0.9700
S1—C1	1.747 (2)	C7—C11	1.495 (4)
N1—C1	1.289 (3)	C7—C8	1.530 (4)
N1—N2	1.388 (3)	C7—H7	0.9800
N2—C2	1.313 (3)	C8—H8A	0.9700
N3—C2	1.346 (3)	C8—H8B	0.9700
N3—C13	1.437 (3)	C9—C10	1.538 (4)
N3—H3	0.872 (9)	C9—H9A	0.9700
C1—C3	1.500 (3)	C9—H9B	0.9700
C3—C4	1.530 (3)	C10—C11	1.513 (4)
C3—C9	1.532 (3)	C10—C12	1.518 (4)
C3—C8	1.545 (3)	C10—H10	0.9800
C4—C5	1.535 (4)	C11—H11A	0.9700
C4—H4A	0.9700	C11—H11B	0.9700
C4—H4B	0.9700	C12—H12A	0.9700
C5—C12	1.511 (4)	C12—H12B	0.9700
C5—C6	1.514 (4)	C13—H13A	0.9600
C5—H5	0.9800	C13—H13B	0.9600
C6—C7	1.509 (4)	C13—H13C	0.9600
C6—H6A	0.9700		
			
C2—S1—C1	87.21 (11)	C11—C7—H7	109.5
C1—N1—N2	114.59 (18)	C6—C7—H7	109.5
C2—N2—N1	111.34 (18)	C8—C7—H7	109.5
C2—N3—C13	119.4 (2)	C7—C8—C3	110.6 (2)
C2—N3—H3	117.1 (16)	C7—C8—H8A	109.5
C13—N3—H3	120.7 (16)	C3—C8—H8A	109.5
N1—C1—C3	125.1 (2)	C7—C8—H8B	109.5
N1—C1—S1	112.82 (18)	C3—C8—H8B	109.5
C3—C1—S1	122.04 (16)	H8A—C8—H8B	108.1
N2—C2—N3	123.8 (2)	C3—C9—C10	110.7 (2)
N2—C2—S1	114.02 (17)	C3—C9—H9A	109.5
N3—C2—S1	122.18 (16)	C10—C9—H9A	109.5
C1—C3—C4	110.38 (18)	C3—C9—H9B	109.5
C1—C3—C9	111.84 (19)	C10—C9—H9B	109.5
C4—C3—C9	108.5 (2)	H9A—C9—H9B	108.1
C1—C3—C8	110.08 (19)	C11—C10—C12	110.7 (2)
C4—C3—C8	108.1 (2)	C11—C10—C9	108.1 (2)
C9—C3—C8	107.8 (2)	C12—C10—C9	108.9 (2)
C3—C4—C5	110.3 (2)	C11—C10—H10	109.7
C3—C4—H4A	109.6	C12—C10—H10	109.7
C5—C4—H4A	109.6	C9—C10—H10	109.7
C3—C4—H4B	109.6	C7—C11—C10	110.1 (2)
C5—C4—H4B	109.6	C7—C11—H11A	109.6
H4A—C4—H4B	108.1	C10—C11—H11A	109.6
C12—C5—C6	109.6 (2)	C7—C11—H11B	109.6
C12—C5—C4	108.4 (3)	C10—C11—H11B	109.6
C6—C5—C4	109.8 (3)	H11A—C11—H11B	108.1
C12—C5—H5	109.7	C5—C12—C10	109.9 (2)
C6—C5—H5	109.7	C5—C12—H12A	109.7
C4—C5—H5	109.7	C10—C12—H12A	109.7
C7—C6—C5	110.5 (2)	C5—C12—H12B	109.7
C7—C6—H6A	109.6	C10—C12—H12B	109.7
C5—C6—H6A	109.6	H12A—C12—H12B	108.2
C7—C6—H6B	109.6	N3—C13—H13A	109.5
C5—C6—H6B	109.6	N3—C13—H13B	109.5
H6A—C6—H6B	108.1	H13A—C13—H13B	109.5
C11—C7—C6	110.4 (3)	N3—C13—H13C	109.5
C11—C7—C8	109.9 (2)	H13A—C13—H13C	109.5
C6—C7—C8	108.0 (2)	H13B—C13—H13C	109.5
			
C1—N1—N2—C2	−0.7 (3)	C12—C5—C6—C7	−58.7 (3)
N2—N1—C1—C3	−177.1 (2)	C4—C5—C6—C7	60.3 (3)
N2—N1—C1—S1	1.3 (2)	C5—C6—C7—C11	58.9 (3)
C2—S1—C1—N1	−1.21 (18)	C5—C6—C7—C8	−61.2 (3)
C2—S1—C1—C3	177.24 (19)	C11—C7—C8—C3	−59.0 (3)
N1—N2—C2—N3	−179.5 (2)	C6—C7—C8—C3	61.4 (3)
N1—N2—C2—S1	−0.3 (2)	C1—C3—C8—C7	179.5 (2)
C13—N3—C2—N2	−5.0 (4)	C4—C3—C8—C7	−59.9 (3)
C13—N3—C2—S1	175.9 (2)	C9—C3—C8—C7	57.3 (3)
C1—S1—C2—N2	0.84 (18)	C1—C3—C9—C10	179.9 (2)
C1—S1—C2—N3	−180.0 (2)	C4—C3—C9—C10	57.9 (3)
N1—C1—C3—C4	−6.8 (3)	C8—C3—C9—C10	−58.9 (3)
S1—C1—C3—C4	174.97 (17)	C3—C9—C10—C11	61.2 (3)
N1—C1—C3—C9	−127.7 (2)	C3—C9—C10—C12	−59.1 (3)
S1—C1—C3—C9	54.0 (3)	C6—C7—C11—C10	−58.0 (3)
N1—C1—C3—C8	112.5 (2)	C8—C7—C11—C10	60.9 (3)
S1—C1—C3—C8	−65.8 (2)	C12—C10—C11—C7	57.7 (3)
C1—C3—C4—C5	178.1 (2)	C9—C10—C11—C7	−61.5 (3)
C9—C3—C4—C5	−59.0 (3)	C6—C5—C12—C10	57.9 (3)
C8—C3—C4—C5	57.7 (3)	C4—C5—C12—C10	−61.9 (3)
C3—C4—C5—C12	61.2 (3)	C11—C10—C12—C5	−57.8 (3)
C3—C4—C5—C6	−58.5 (3)	C9—C10—C12—C5	61.0 (3)

### Hydrogen-bond geometry (Å, º)

**Table:**

*D*—H···*A*	*D*—H	H···*A*	*D*···*A*	*D*—H···*A*
N3—H3···N1^i^	0.87 (1)	2.15 (1)	3.021 (3)	179 (2)

Symmetry code: (i) *x*−1/2, −*y*+3/2, *z*−1/2.
